# Extinction Risk Assessment of the Greek Endemic Flora

**DOI:** 10.3390/biology10030195

**Published:** 2021-03-04

**Authors:** Konstantinos Kougioumoutzis, Ioannis P. Kokkoris, Maria Panitsa, Arne Strid, Panayotis Dimopoulos

**Affiliations:** 1 Laboratory of Botany, Department of Biology, Division of Plant Biology, University of Patras, 26504 Patras, Greece; ipkokkoris@upatras.gr (I.P.K.); mpanitsa@upatras.gr (M.P.); pdimopoulos@upatras.gr (P.D.); 2 Department of Ecology and Systematics, Faculty of Biology, National and Kapodistrian University of Athens, Panepistimiopolis, 15701 Athens, Greece; 3 Bakkevej 6, DK-5853 Ørbæk, Denmark; arne.strid@youmail.dk

**Keywords:** biodiversity conservation, conservation prioritization, GIS analysis, EDGE, EU Biodiversity Strategy, Natura 2000

## Abstract

**Simple Summary:**

This study assesses for the first time all the vascular endemic plant taxa of Greece, according to their decline and rarity. Phylogenetic analysis and its spatial overview highlight areas for conservation prioritization. Several of the Greek endemics are threatened with extinction and fourteen of them need to be prioritized, due to their evolutionary distinctiveness. This assessment could act as the baseline and supporting tool for conservation actions, decision- and policy-making for biodiversity, while highlighting the need for a new Red Data Book for the Greek flora.

**Abstract:**

Human-induced biodiversity decline has been on the rise for the past 250 years, due to various causes. What is equally troubling, is that we are unaware which plants are threatened and where they occur. Thus, we are far from reaching Aichi Biodiversity Target 2, i.e., assessing the extinction risk of most species. To that end, based on an extensive occurrence dataset, we performed an extinction risk assessment according to the IUCN Criteria A and B for all the endemic plant taxa occurring in Greece, one of the most biodiverse countries in Europe, in a phylogenetically-informed framework and identified the areas needing conservation prioritization. Several of the Greek endemics are threatened with extinction and fourteen endemics need to be prioritized, as they are evolutionary distinct and globally endangered. Mt. Gramos is identified as the most important conservation hotspot in Greece. However, a significant portion of the identified conservation hotspots is not included in any designated Greek protected area, meaning that the Greek protected areas network might need to be at least partially redesigned. In the Anthropocene era, where climate and land-use change are projected to alter biodiversity patterns and may force many species to extinction, our assessment provides the baseline for future conservation research, ecosystem services maintenance, and might prove crucial for the timely, systematic and effective aversion of plant extinctions in Greece.

## 1. Introduction

According to recent estimates, our planet hosts up to 430,000 plant species [1], with nearly 2000 taxa being described each year [2]. This remarkable plant diversity is unevenly distributed [3], as a result of geo-historical processes and environmental filtering, with few countries hosting >1000 endemic species [4]. Several areas act as global biodiversity hotspots [5,6] and are facing intense anthropogenic pressure [7]. Even though extinction is the unavoidable fate of every taxon that has ever existed, the background extinction rate is well below the one currently observed [7,8,9], irrespective of the taxon considered [10]. Human-induced biodiversity decline has been on the rise for the past 250 years, due to various causes (e.g., urbanization, agricultural intensification, biotic invasions, habitat degradation/loss) that exhibit clear spatiotemporal patterns [7], leading to biotic homogenization at all biodiversity levels (alpha, beta and gamma) [11,12,13,14] and subsequently to a quantitative alteration or decline of ecosystem services. Even though plants are extinction-resilient [15], current extinction rates reach up to 1.26 extinctions per year [7], thus forcing conservationists to set a maximum desired extinction threshold to incite the post-2020 biodiversity policy agenda [16].

Nearly a decade ago, the Convention on Biological Diversity (CBD) established the ambitious Aichi Biodiversity Targets, the most prominent of which being targets 2, 11, and 12 calling for (i) an extensive risk assessment of most plant species, (ii) establishing a minimum threshold of the percentage of terrestrial land under some form of protection, and (iii) averting the extinction of known threatened species, respectively [17]. Nevertheless, as the recent CBD assessment states, current conservation strategies seem rather ineffective in preventing biodiversity decline. It is becoming increasingly evident that the Aichi Biodiversity Targets will hardly be met [18] and the most worrying fact is that we are unaware which plants are threatened and where these plants occur [19]. This phenomenon may be attributed to several factors, such as the biased taxonomic coverage of the IUCN Red List (ca. 10% of plant species have been assessed—[20,21,22]), the Linnean and Wallacean shortfalls [23,24], the limited economic resources ([21] and references therein), the CBD’s ‘soft law’ approach [21], as well as to the fact that IUCN extinction risk assessments—the global golden standard for assessing extinction risk [25,26]—are time-consuming and resource-intensive ([21,27] and references therein). Consequently, this situation has led to calls for cost-effective methods that speed-up the extinction risk assessment process ([27,28,29,30] and references therein) and this is even more needed now, taking into consideration the increasing intensity of anthropogenic threats to biodiversity over the coming decades [7]. Such proactive approaches minimize long-term extinction risk, while being economically efficient [31,32].

Aichi Biodiversity Target 2 is the foundation stone of any conservation project/prioritization scheme, since once a thorough extinction risk assessment has been performed and the spatial patterns of threat have been revealed [19,21], we can allocate the available funds for the protection of those species most in need and devise a cost-effective management plan optimizing species conservation [33], thus overcoming the ‘Anthropocene vs Ecocentrism’ debate ([19] and references therein). By doing so, we may move forward towards Aichi Biodiversity Targets 5, 7 and 8 (protecting important areas for plant diversity, in- and ex-situ conservation of most threatened plant species, respectively). The incorporation of phylogenetic diversity metrics, such as the Evolutionary Distinct and Globally Endangered (EDGE) index, a geographically and threat-weighted variant of phylogenetic diversity [34,35], has been proved useful when defining and prioritizing important plant diversity areas [34], as it complements Aichi Biodiversity Target 2.

**Figure 1:**
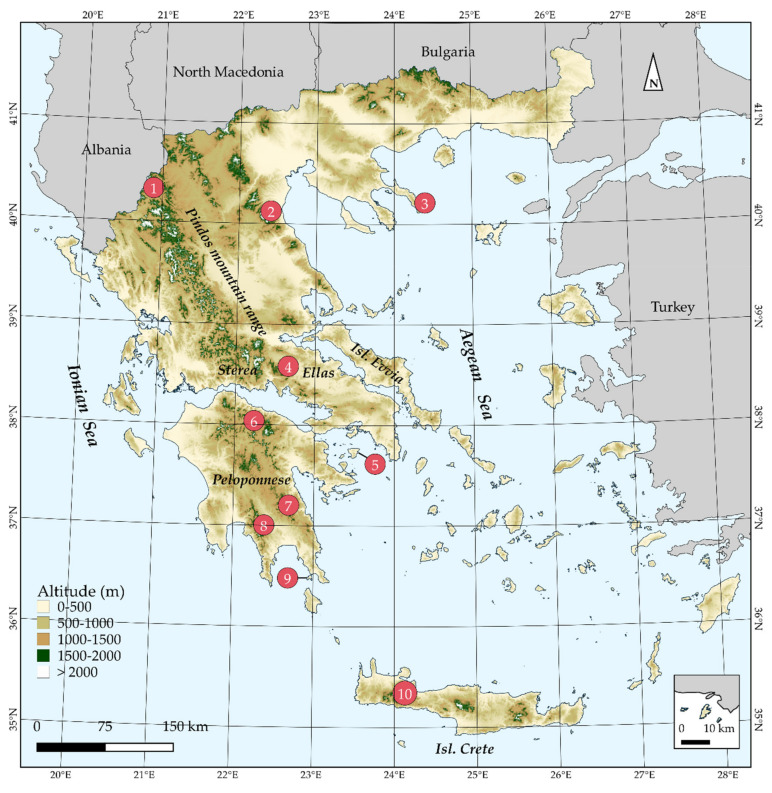
Map of Greece presenting major mountain massifs, as well as Aegean islands mentioned in the text. 1: Mt. Gramos, 2: Mt Olymbos, 3: Mt Athos, 4: Mt Parnassos, 5: Isl Aegina, 6: Mt Chelmos, 7: Mt Parnonas, 8: Mt Taygetos, 9: Isl Elafonisos, 10: Lefka Ori mountain range. The inset map depicts the island of Megisti (Kastelorizo) and its nearby islets.

The extinction risk assessment of endemic plant species under the IUCN standards and Criteria constitutes the most objective and rigorous prioritization conservation and dissemination medium among conservationists, practitioners, and decision-makers [25,26]. However, very few countries, irrespective of their economic prosperity or plant species richness, have assessed in its entirety their endemic flora [4], despite the availability of cost-effective, rapid, reliable and automated conservation assessment methods [28,36,37]. Greece (Figure 1), one of the most biodiversity-rich (>7000 native plant taxa) and environmentally heterogeneous countries in Europe and the Mediterranean Basin [38], has not departed from this principle, as has yet to assess its >1400 endemic plants [4]. Greece hosts ca. 40% of the plant taxa assessed under the IUCN standards at the European level, with 65 taxa rendered as threatened (https://www.iucn.org/sites/dev/files/content/documents/greece_s_biodiversity_at_risk_fact_sheet_may_2013.pdf (accessed on 3 September 2020)). Nonetheless, no comprehensive extinction risk assessment that complies with the IUCN Criteria exists for the Greek endemic plants up to this day, even though two such attempts have been made for a fraction of the Greek flora in the past 25 years [39,40]. However, these attempts are now outdated based on the IUCN standards (>10 years have passed since the latest Greek Red Data Book). Despite failing to meet Aichi Biodiversity Target 2, Greece has done exceptionally well in reaching Aichi Biodiversity Target 11, as the established Natura 2000 network covers up to 28% of the terrestrial territory of Greece [41]. The Linnean and Wallacean shortfalls (the taxonomic and geographical distribution knowledge gap, respectively; [42]) in Greece have been readily addressed as well [43], due to the long-lasting interest of many botanists and biogeographers operating in the region during the last two centuries ([44,45,46] and references therein). Finally, recent advances regarding the Darwinian shortfall (the phylogenetic relationships knowledge gap; [42]) [47,48] enable the assimilation of phylogenetic diversity metrics into conservation evaluations. Therefore, the road has been paved to perform an extinction risk assessment for all the endemic plant species occurring in Greece and, thus, move a step closer to the fulfilment of the country’s obligation regarding Aichi Biodiversity Target 2, and thereupon Aichi Biodiversity Targets 5 and 12. Our aim is to perform the first phylogenetically-informed, extinction risk assessment for the entire Greek endemic flora and delineate the areas that need to be prioritized in the near future.

## 2. Materials and Methods

**Figure 2:**
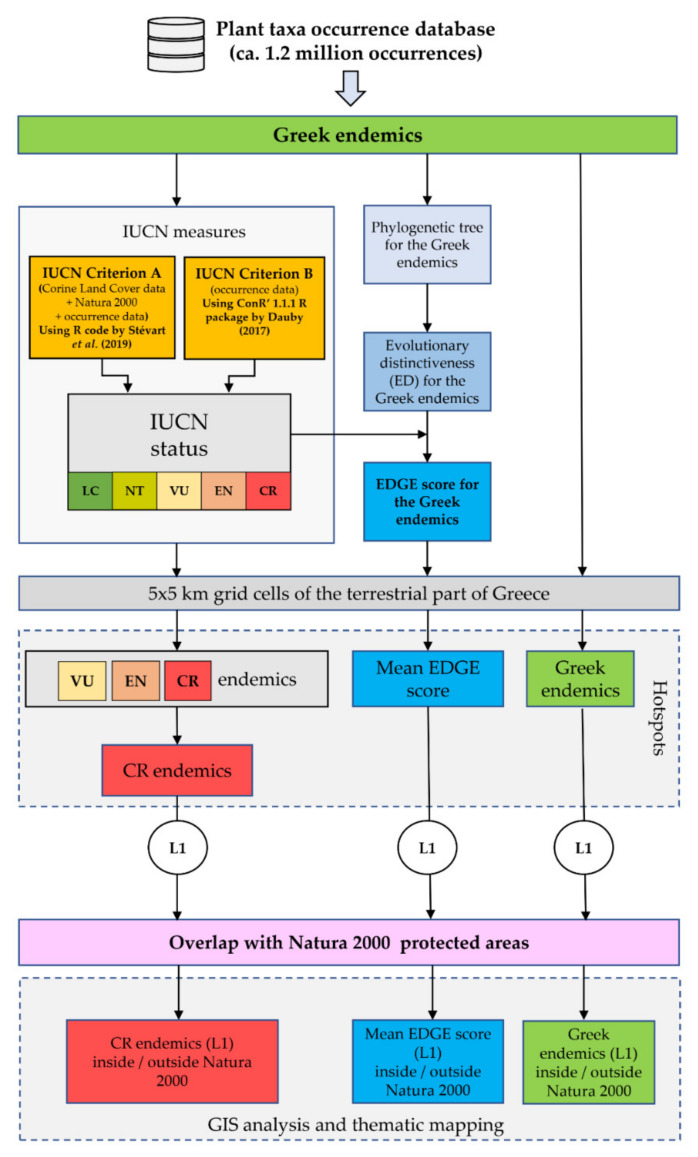
Flowchart of our methodological workflow. EDGE: Evolutionary Distinct and Globally Endangered. L1 hotspots: the 1% of cells (i.e., the 1% quantile) that had the highest score for each metric. CR: Critically Endangered. EN: Endangered. VU: Vulnerable. LC: Least Concern. NT: Near Threatened.

The methodological workflow is analytically described in the following subsections and it is graphically presented in Figure 2. The first and main part of the workflow assesses the extinction risk status for each Greek endemic taxon based on the IUCN Criteria A and B in a cost-effective, fast and robust framework (using R code from [28,36]), estimates their evolutionary distinctiveness and calculates their EDGE score. The second part of the workflow identifies the areas with the highest threatened species richness and EDGE score, while, as a final step, we calculate the overlap of these areas with the Greek protected areas network. We should note that our extinction risk assessments are not based on the IUCN Criteria C and D, as detailed population-level data for the entire Greek endemic flora do not exist (the same trend is observed for IUCN assessments in general, since only 8.9% of them rely on Criteria C or D; [27]).

### 2.1. Species Occurrence Data

Greece hosts 7043 native plant taxa (species and subspecies), 1435 of which are Greek endemics (GR—[38,49]). Following [38,49], taxa are defined as (a) subspecies and (b) species that have no subspecies, i.e., when a species has subspecies, then only its subspecies are counted. Our final dataset comprises 1384 Greek endemic taxa, since 51 species include one or more subspecies. All subsequent analyses are based on the most extensive and detailed database (Flora Hellenica Database, Strid (ongoing)) of plants occurring in Greece (~1.2 M occurrences). All plant taxa were cross-checked for synonyms, following the nomenclature proposed by [38,49]. To locate the areas with the highest endemic richness, we calculated the number of Greek endemic taxa occurring at a 5 × 5 km grid cell using QGIS 3.14 [50], following [51]. 

### 2.2. IUCN Measures

We assigned each Greek endemic taxon to an IUCN threat category according to the IUCN Criteria A and B using the R code provided by [28] and the ‘ConR’ 1.1.1 R package [52], respectively. ConR is a fast, highly sensitive and accurate method for performing extinction risk assessments [37,53,54]. It calculates the standard IUCN measures Extent of Occurrence (EOO) and Area of Occupancy (AOO), as well as the number of locations for every taxon based on occurrence records following the IUCN recommendations [55] for Criterion B, the most widely applied criterion for official extinction risk assessments [56]. Regarding Criterion A, the approach of [28] estimates potential population reduction using occurrence data based on land-cover classes characterized by moderate-to-high human influence which are directly linked to the main threats of the study taxa, as population-level data are rarely available for plants [57]. By doing so, the potential decline in habitat quality for each taxon can be identified and thus the potential population reduction can be inferred using each taxon’s potential decrease in AOO [28]. To calculate Criterion A under this approach, we used the CORINE land cover (CLC) data v.20 (https://land.copernicus.eu/pan-european/corine-land-cover/clc2018?tab=download (accessed on 3 September 2020)). CLC layers 1 (artificial areas) and 2 (agricultural areas—apart from 223 (olive groves) and 243–244 (land principally occupied by agriculture, with significant areas of natural vegetation and agro-forestry areas, respectively)) are directly linked to the main threats on Mediterranean and Greek endemic taxa [58,59,60,61,62,63]. Olive groves were not included in the CLC threat layers, as they represent (semi-)natural ecosystems not directly linked to the main threats on Greek endemics [60,63]. 

After Greek endemic taxa were assigned to an IUCN category, we estimated the total number of taxa recorded and the proportion of taxa assessed under each IUCN category, under Criteria A and B separately, and by combining both criteria, i.e., a Greek endemic taxon would, for example, be categorized as CR (CR_END_) if it is assessed as CR by at least one of the two criteria. We should note that this approach does not substitute full Red List assessments, but in the absence of such official, time-consuming and resource-intensive assessments [27], it provides a fast, robust and reliable alternative using the two most commonly applied IUCN criteria [22,56,57,64] (besides, Criteria C and D can be considered as special cases of Criteria A and B: the sub-criteria for Criterion C rely on population reduction as in Criterion A and Criterion D2 relies on the estimated AOO as in Criterion B [57]) following the IUCN guidelines, that may serve as a baseline for more in-depth conservation assessments in the future [28,53,54], while also contributing towards Aichi Biodiversity Target 2 and assisting in effective, evidence-based conservation decision making [65].

We aggregated the IUCN threat categories into two (threatened (CR, EN, VU) and not threatened (LC, NT)) to estimate the accuracy (overall, how often was the classifier correct when predicting threat categories) and sensitivity (true positive rate) [66,67,68,69,70] of the extinction risk assessments based on Criteria A and B via a confusion matrix, using functions from the ‘caret’ 6.0-86 R package [71] following [37,53]. As a reference dataset we used the Greek endemics that have been previously been assessed according to the IUCN Criteria [39,40] and are not data-deficient (n = 238).

We calculated the number of Greek endemic taxa occurring in each grid cell for every IUCN category. We defined GR, CR_END_ and EDGE hotspots as the 1% of cells (i.e., the 1% quantile; L1 hotspots) that had the highest score for each metric, following [72]. Biodiversity hotspots are herein and hereafter defined as local biodiversity hotspots (i.e., hotspots within a regional biodiversity hotspot, which is part of a global biodiversity hotspot—[73]).

We did not extend our analyses to the native non-endemic taxa occurring in Greece, since regional IUCN assessments (i.e., assessments not restricted to endemic taxa) may inaccurately estimate the extinction risk of the native non-endemic taxa whose (sub-)populations are defined by geopolitical borders [74] and, thus, run the risk of not being considered for inclusion in the IUCN Red List (https://www.iucnredlist.org/assessment/process (accessed on 3 September 2020)).

### 2.3. Estimation of the EDGE Index

For the estimation of Evolutionary distinctiveness (ED) we used the time-calibrated tree from [51], keeping only the Greek endemics (GR). Evolutionary distinctiveness was then calculated for the Greek endemics in the time-calibrated tree using the ‘picante’ 1.6.2 [75] R package. EDGE scores were calculated using the formula given in [35]:

(1) $$\mathrm{EDGE}\;=\ln\left(1\;+\mathrm{ED}\right)+\;\mathrm{GE}\times \ln\left(2\right)$$

where ED is the ED value of a taxon as calculated in ‘picante’ and GE is its weighted IUCN threat category [LC = 0; NT = 1; VU = 2; EN = 3; CR = 4] on a log scale. EDGE scores represent the log-transformed taxon-specific anticipated loss of evolutionary history, in which each increase of one Red List category constitutes a two-fold increase of extinction risk [35].

We then derived the mean EDGE values for the Greek endemics present in each grid cell.

### 2.4. Protected Areas Network Overlap

We overlapped L1 hotspot results with the protected areas (PAs) network retrieved from the World Database on Protected Areas (WPDA) using GIS-related functions from the “wdpar” 1.0.0 [76] and the “sf” 0.8.0 [77] R packages. The overlap analysis is limited to the terrestrial part of the country.

## 3. Results

### 3.1. IUCN Measures

**Figure 3:**
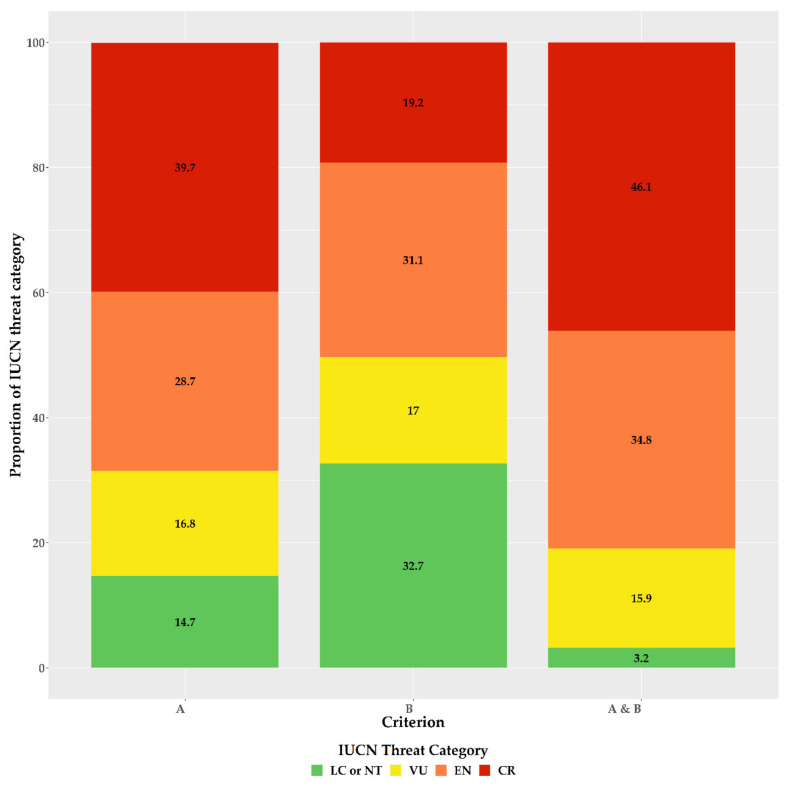
Proportion of the Greek endemic taxa under the IUCN threat categories according to Criterion A, Criterion B, and both Criterion A and B (from left to right).

**Figure 4:**
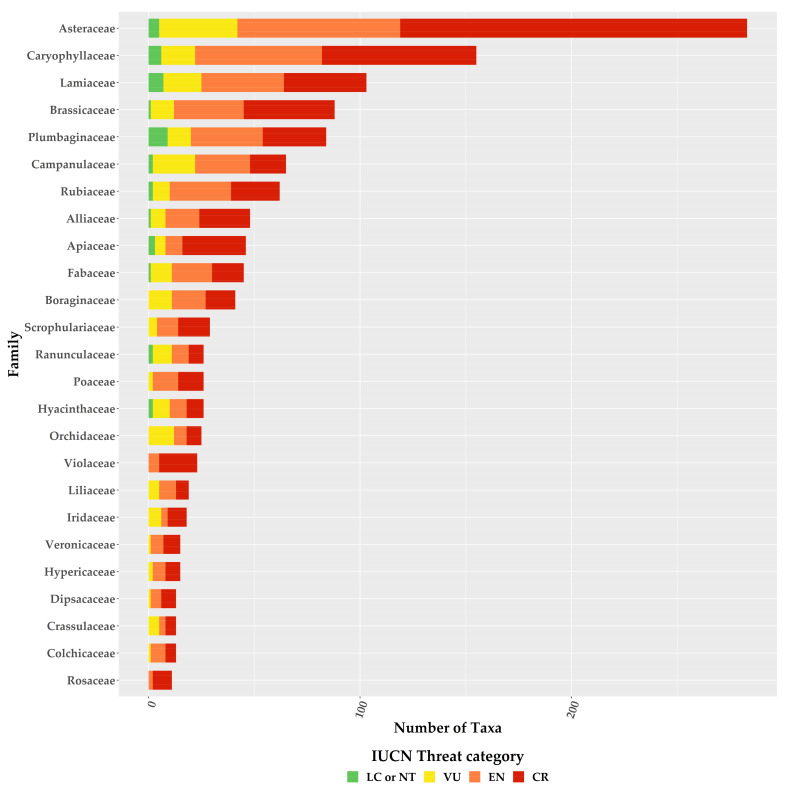
Proportion of the Greek endemic taxa under the IUCN threat categories according to both Criterion A and B for the richest plant families. Please note that information is presented only for families with 10 or more Greek endemic taxa.

**Figure 5:**
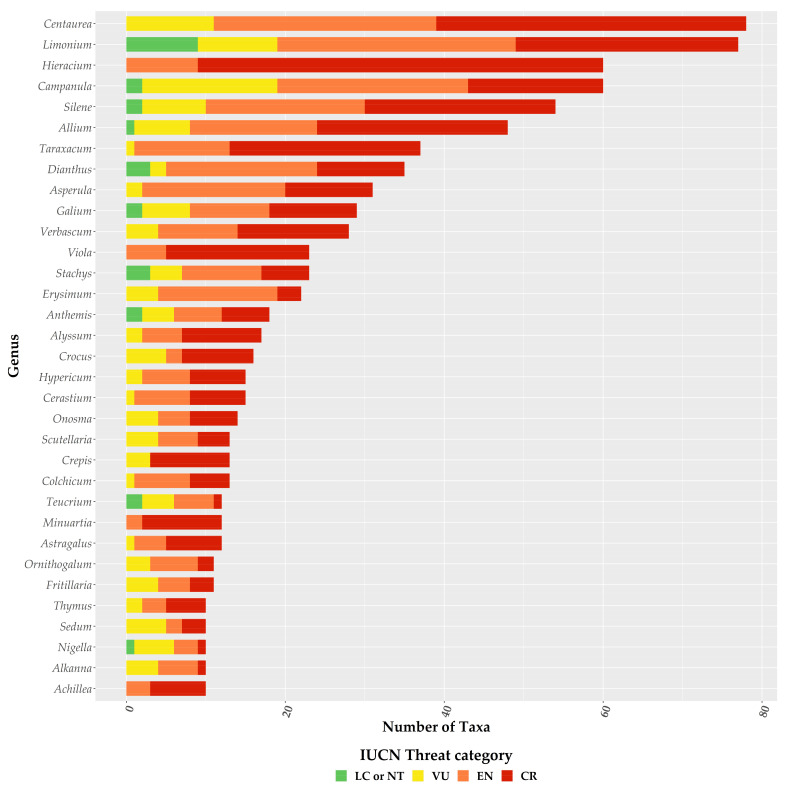
Proportion of the Greek endemic taxa under the IUCN threat categories according to both Criterion A and B for the richest plant genera. Please note that information is presented only for genera with 10 or more Greek endemic taxa.

For the first time, we provide an assessment of every Greek endemic taxon according to the IUCN Criteria A and B (Figure 3, Figure 4 and Figure 5; Supplementary Materials Table S1). At present, 46.1% of Greek endemics are facing imminent extinction and are considered as Critically Endangered (CR—Figure 3) according to both Criteria A and B, while 39.7% and 19.2% are characterised as CR under the Criterion A and B, respectively (Figure 3). Asteraceae, Caryophyllaceae, and Brassicaceae have the most taxa identified as CR (Figure 4; Supplementary Materials Table S1), while Violaceae have the highest percentage of CR taxa (if a family has 10 or more CR taxa; Supplementary Materials Table S2). At the genus level, *Hieracium* and *Minuartia* show the highest percentage of CR taxa (85.0% and 83.3%, respectively—Supplementary Materials Table S3). As for the genera with the most CR taxa, *Hieracium*, *Centaurea*, and *Limonium* have the most taxa identified as CR (if a genus has 10 or more GR; Figure 5; Supplementary Materials Table S3). All threat assessments for the CR taxa are available in Supplementary Materials Table S4.

Accuracy and sensitivity ranged between 65.1–82.4% and 79.1–98.0%, respectively, depending on the criterion used (Supplementary Materials Table S5), with threat assessments based on the Criterion B showing the highest accuracy. When both criteria were applied, accuracy and sensitivity were 80.7% and 98.0%, respectively (Supplementary Materials Table S5).

Based on both Criteria A and B, the Greek endemics have EDGE scores in the range 1.66–8.77, with most taxa (75%) falling in an EDGE score class of 1.66–4.95 (Supplementary Materials Table S1 and Figure S1). Fourteen taxa have an EDGE score exceeding 7.0, belonging to eleven different families and thirteen different genera, all being angiosperms, except for three taxa (*Abies cephalonica*, *Asplenium creticum*, and *Isoetes heldreichii*, Supplementary Materials Table S6); only *Abies cephalonica* is not considered as a CR taxon.

### 3.2. Current Spatial EDGE and Threat Patterns

**Figure 6:**
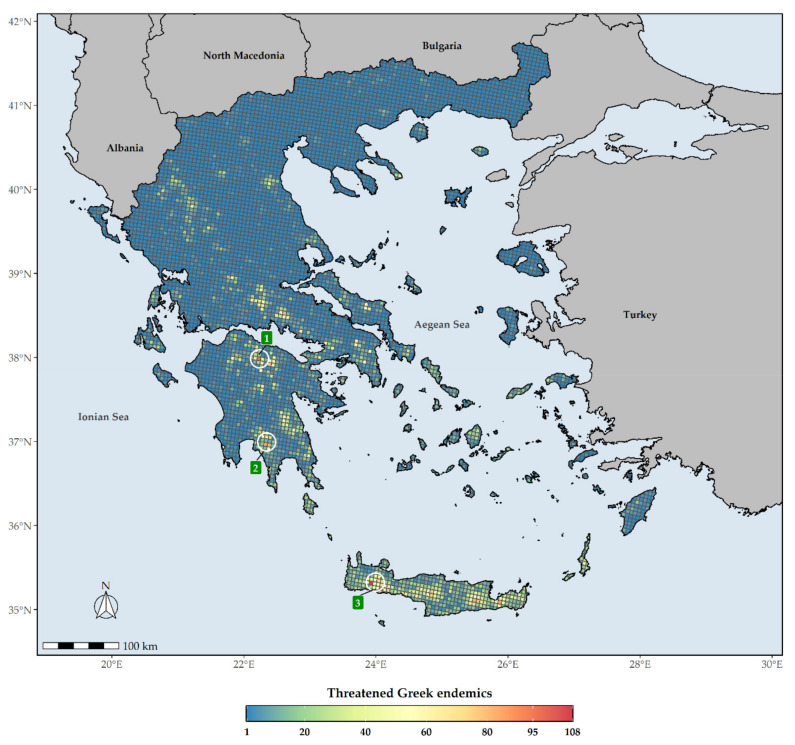
Species richness in Greece regarding threatened Greek endemic taxa (GR) for every grid cell in Greece. Grid cell resolution equals to ca. 5 km. 1: Mt. Chelmos, 2: Mt. Taygetos, 3: Lefka Ori mountain range. The white circles delineate (roughly) the aforementioned mountains and mountain ranges.

**Figure 7:**
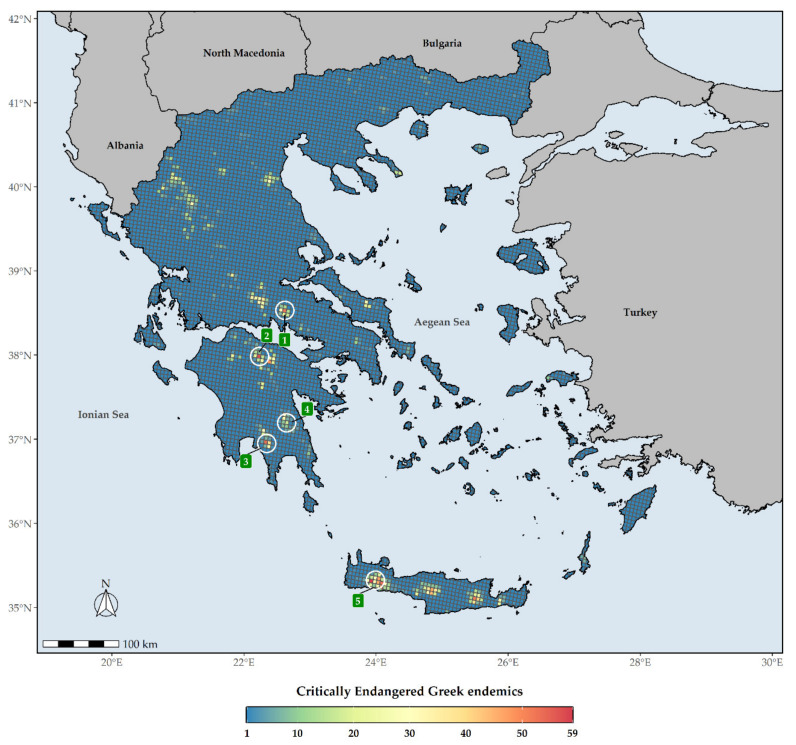
Species richness in Greece regarding Critically Endangered Greek endemic taxa (CR_END_) for every grid cell in Greece. Grid cell resolution equals to ca. 5 km. 1: Mt. Parnassos, 2: Mt. Chelmos, 3: Mt. Taygetos, 4: Mt. Parnonas, 5: Lefka Ori mountain range. The white circles delineate (roughly) the aforementioned mountains and mountain ranges.

The distribution of the Greek endemic taxa assessed as threatened under the IUCN categories under both Criteria A and B are not uniform across Greece (Figure 6). These taxa are mostly concentrated at the Cretan and Peloponnesian (i.e., Mts. Chelmos and Taygetos) mountain massifs, with the highest number of them occurring in Lefka Ori mountain range in Crete (Figure 6). As for the CR_END_ taxa, these are mainly found on the Cretan mountain massifs, Mt. Parnassos in Sterea Ellas and Mt. Chelmos and Mt. Taygetos in the Peloponnese (Figure 7). L1 hotspots for CR_END_ and GR largely coincide, with most L1 hotspots occurring in Crete and the Peloponnesian mountain massifs (Supplementary Materials Figures S2 and S3), the main difference being that the northern Pindos mountain range and Mt. Olymbos constitute L1 CR_END_ hotspots, but not L1 GR hotspots.

**Figure 8:**
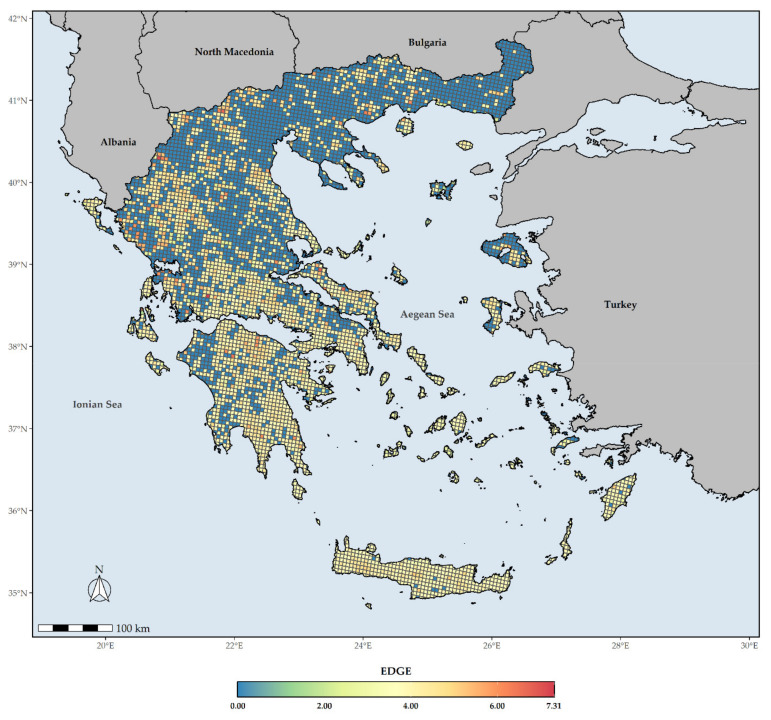
The mean EDGE index score for every grid cell in Greece. Grid cell resolution equals to ca. 5 km.

**Figure 9:**
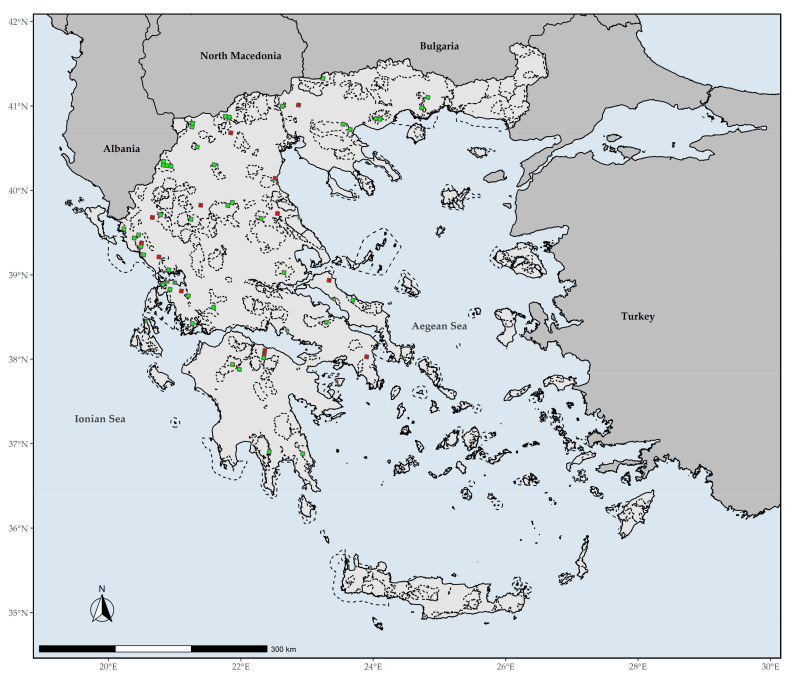
Red and green coloring indicates grid cells with L1 (top 1%) EDGE values that fall outside or inside the Greek protected areas, respectively. Dashed lines denote the protected areas present in Greece.

The EDGE index spatial patterns show that large parts of Southern Greece are currently identified as hosting assemblages of great evolutionary distinctiveness facing immediate extinction risk (Figure 8). However, the areas with very high values of the EDGE index are currently found in the wider area around Mt. Gramos. Only six L1 EDGE hotspots do not occur in the mainland (i.e., the western part of Elafonisos, Aegina, and NE and C Evvia), while most L1 EDGE hotspots occur in Western Greece and the mainland mountain massifs (Figure 9).

### 3.3. Protected Areas Network Overlap

The overlap with the Greek PAs revealed that all L1 CR_END_ and GR hotspots are within the PA network (Supplementary Materials Figures S2 and S3). On the other hand, 27.5% of the L1 EDGE hotspots fall outside PAs network in Greece (Figure 9).

## 4. Discussion

According to recent estimates, ca. 40% of vascular plants are facing extinction [22], yet the extinction threat status of most known plants remains still unknown, as well as where these threatened plant taxa occur [19]. This hinders our efforts to efficiently conserve and protect the taxa and areas most at risk [78] and calls for an increase in extinction risk assessments under the IUCN criteria [20,21].

To this day, a fraction (17.83%—n = 238) of the Greek endemics has been assessed under the IUCN criteria, most of them being local mountain or island endemics [39,40]. This detailed, yet limited knowledge in quantitative terms (considering the large number of Greek endemic taxa), funneled the conservation efforts to specific areas of the country to protect and/or improve the conservation status of the aforementioned taxa, by e.g., delineating protected areas to include their entire distribution, thus lowering the chances of implementing a nation-wide, effective conservation management scheme [79]. As [39] state, they did not provide a comprehensive, nor complete assessment of the Greek endemic flora ([39], p. XXIII). They also consider the threatened species number and their assessment as dynamic and not static ([39], p. 23), concluding that “knowledge is power” [39,40]. Inspired by [39,40], we conducted the first ever phylogenetically-informed assessment of the extinction threat status of the entire Greek endemic flora based on the IUCN criteria A and B and uncovered for the first time the threat distribution patterns and hotspots across the Greek territory. Thus, we are able to: (i) identify the taxa that are in urgent need of conservation attention (i.e., those with high EDGE scores), (ii) suggest the taxa entailing a full Red List assessment (e.g., as in Spain—[80]), and (iii) propose that a revision is needed regarding the national conservation strategy, since the threat distribution patterns we unveiled are entirely different from the perceived conservation reality in Greece. More specifically regarding point (iii), a significant portion of the identified conservation hotspots is not included in any designated Greek protected area, meaning that the Greek protected areas network might need to be at least partially redesigned, as suggested also by [51,78].

### 4.1. The Greek Flora under Threat

In order to meet Aichi Biodiversity Target 2, an accurate and updated priority list is needed and even more so, for countries especially rich in endemics (>1000 taxa), such as Greece, which hosts 1435 endemic plant taxa, amounting to 20.4% of its native flora [38,49]. Assessing their extinction risk is therefore important for the fulfilment of the country’s obligation regarding Aichi Biodiversity Target 2. Herein, we provide the largest conservation assessment of the endemic vascular flora of a given country in the Mediterranean and the European Union (EU) that could serve as the plant conservation basis for the Mediterranean and the EU (e.g., contribute to the European and Mediterranean Regional Assessment initiatives by IUCN; https://www.iucnredlist.org/regions/europe (accessed on 3 September 2020)); https://www.iucnredlist.org/regions/mediterranean (accessed on 3 September 2020)), in conjunction with the results obtained for Italy [79] and Spain [80].

Our assessment’s accuracy and sensitivity (80.7% and 98.0%, respectively) is in line with global and regional estimates [37,53,54,65] and can be thus considered reliable and robust, as it agrees with the findings of [39,40], regarding the taxa previously assessed (n = 238). We found that the vast majority of the Greek endemics are considered as threatened (Figure 3), which is in harmony with the previous and now outdated assessments conducted in Greece (Greek endemic taxa assessed: n = 238; ca. 85% of the taxa assessed were threatened; [39,40]). Based on both IUCN Criteria A and B, nearly half (46.1%) of the Greek endemics are facing imminent extinction (Figure 3), which is in line with the most recent global estimations [22]. This proportion is much higher than the estimates from Italy (22.4—[79]) and Spain (22.1%—[80]), two other highly biodiverse Mediterranean countries, which can be ascribed to the fact that the extinction risk of the Italian and Spanish endemics was based almost entirely only on Criterion B [79,80]. Taking into consideration the extinction risk for the Greek endemics based only on the latter criterion, then 19.2% of the Greek endemics are facing imminent extinction (Figure 3), which is roughly the same with that reported from Italy and Spain. Either way, this sets on the alarm for revising national conservation priorities, since endemic taxa represent an invaluable resource in terms of genetic diversity and constitute key elements of particular importance at local, regional, national and global level [81,82], as their survival is entirely dependent on national responsibility and the relevant policies and practice [79]. We recorded an elevated extinction risk for some taxon-rich genera, such as *Allium*, *Centaurea*, *Hieracium*, and *Limonium*, for which Greece represents their diversification or diversity center (for at least some of their sections—e.g., [83,84]). The majority of these taxa occur either in coastal or lowland areas, where land-use change due to human activities has been intensifying during the past two decades (https://land.copernicus.eu/pan-european/corine-land-cover (accessed on 3 September 2020)); [85,86]) leading to habitat loss and degradation, two major factors related to increased extinction rates [7,12,22], especially in the Mediterranean [85]. Currently, only one taxon (*Isoetes heldreichii*) is presumably extinct in Greece [87], while other taxa that were considered extinct, were recently rediscovered (e.g., [88,89]). The recorded extinction rate in Greece is thus lower than Italy [79], Spain [90], other areas with Mediterranean-type climate (e.g., [91]) or global estimates [7,9,22], and cannot be attributed to lower collection effort, since Greece has been extensively botanized during the past two centuries [92], with collection intensity intensifying the past decades, which resulted in the rediscovery of many presumably extinct taxa [92]. The lower extinction rates observed in Greece might be due to extinction debt (i.e., the delayed extinction of taxa—[93]), as a result of the lower industrialization and urbanization rates of Greece compared to Italy and Spain [94,95] until the 1960s [96]. This situation may however change dramatically in the foreseeable future, since most Greek endemics are either very narrowly-distributed (Supplementary Materials Table S1) or occur in areas highly affected by human activities ([38,49]—Figure 3). Another key factor that may contribute to an increased extinction rate in Greece, is the inability of a significant portion of the Greek endemics to track the shift of their realized niche in a changing climate, as exemplified by the single island endemics of Crete [97] or other rare plant taxa occurring in Greece (e.g., [98,99,100,101]).

By incorporating phylogenetic diversity (i.e., the EDGE index) into conservation analyses, we are able to define and prioritize important plant diversity areas where the intersection between different facets of biodiversity (taxonomic, phylogenetic) is high and assess the efficiency of the currently established PA networks [34,102,103]. The majority of the fourteen Greek endemic taxa that had a very high EDGE score (Supplementary Materials Table S6) and are, thus, of high conservation priority, have already been included in conservation management and monitoring projects, and implemented by the respective Management Body in which they occur. On the one hand, this means that our analyses were able to properly identify the taxa most at risk nationwide and on the other hand, that the Greek conservation agencies have been correctly and efficiently allocating their limited funds, despite the lack of national structural strength and the economic austerity [104].

The Cretan and Peloponnesian mountain massifs constitute threatened Greek endemic diversity hotspots (Figure 6), with the wider area around Mt. Gramos having the highest EDGE score, thus qualifying as the most important conservation hotspot in Greece (Figure 8). It is worth mentioning that even though the Cretan mountain massifs host the most threatened/CR Greek endemic taxa (Figure 6 and Figure 7), they do not constitute L1 EDGE hotspots (Figure 9), probably an incidence of the non-adaptive radiations (evolutionary diversification from a single ancestor, not accompanied by niche differentiation [105]) of the Cretan single island endemics (a prominent example being the *Dianthus juniperinus* complex or the *Campanula*/*Roucela* taxon complex). Even though several areas that are renowned for their outstanding floristic uniqueness and plant species richness (e.g., the Pindos mountain range, Mt. Olymbos, Mt. Athos, Mt. Parnassos, Mts. Chelmos, Taygetos and Parnonas in the Peloponnese, the Cretan mountain massifs, as well as the mountains of Evvia [51]) are rendered as extinction risk hotspots (Figure 6, Figure 7 and Figure 8), the spatial distribution of threatened Greek endemic taxa all over the Greek territory, inside and outside the protected areas’ network (Figure 3; Supplementary Materials Figures S2 and S3), imply that a general strategic plan should be developed at the national scale. The ongoing, national Life Integrated Project for ‘Integrated actions for the conservation and management of Natura 2000 sites, species, habitats and ecosystems in Greece’ (https://ec.europa.eu/environment/life/project/Projects/index.cfm?fuseaction=search.dspPage&n_proj_id=6520 (accessed on 3 September 2020)) can be substantially supported by these results and integrate them into the proposed National Set of MAES (Mapping and Assessment of Ecosystems and their Services) Indicators (e.g., the indicators of endemic diversity—[106]) assessments [41] for ecosystem condition and plant diversity. Subsequently, our results provide crucial information for the natural capital assessment and accounting process in Greece, supporting state efforts to follow the EU Biodiversity Strategy and EU Green Deal guidelines and fulfil relevant targets.

### 4.2. The Role of Protected Areas

In Greece, the Natura 2000 protected areas network covers almost the total spatial distribution of the Greek endemic taxa assessed as Threatened and/or having high EDGE index score. However, until today all conservation efforts for plant taxa within Natura 2000 sites are targeted on Dir. 92/43/EC taxa. In National Parks more taxa are taken into account: mainly local endemics or taxa assessed as threatened by previous surveys. Most of these efforts are limited to monitoring assessments under EU obligations for Article 17 reporting. Relevant conservation actions concern institutional measures of prohibition and/or local implementation actions for the improvement of their populations and habitats. Here, we document and highlight a general overlap among areas with a high number of Greek endemic taxa under imminent extinction risk (Supplementary Materials Figure S3), high EDGE score (Figure 8 and Figure 9) and high endemism rate (Supplementary Materials Figure S2), inside the Natura 2000 network (Supplementary Materials Figures S2 and S3). These parts of Natura 2000 sites are considered particularly important, and where conservation efforts and management strategies for plant taxa and their habitats should focus on. However, a significant portion of the L1 EDGE hotspots, i.e., the areas identified as those of immediate conservation concern, is not included in any designated Greek PA, while other PAs do not encompass either a L1 EDGE or CR_END_ or GR hotspot. This might be ascribed to the Linnean, Wallacean, and Darwinian shortfalls at the time that this network was designed and denotes that the Greek PA network might need to be at least partially redesigned, in order to include these L1 EDGE hotspots. This is in line with [51,78], who highlighted the need of expanding the Greek PA network, even though Greece ranks among the top-10 EU countries regarding their PA coverage (http://www.eea.europa.eu/data-and-maps/dashboards/natura-2000-barometer (accessed on 3 September 2020)). After all, the expansion of the existing PAs will be included in the post-2020 conservation agenda in order to protect several threatened taxa [18,107,108]. This can also be achieved by establishing community protected areas or other effective area-based conservation measures [109,110]. Thus, we suggest that the monitoring and conservation targets should be revised and be set further from the country’s obligations for EU reporting to local (site-specific) needs and demand. This implies that the Greek Natura 2000 network is in its vast majority well-structured and spatially delineated, as far as current Greek endemic plant taxa threat status is concerned. The information provided here should also be considered for future assessments especially for environmental licensing of human activities inside the Natura 2000 protected area network and for drafting plant taxa Action Plans (i.e., inclusion in the relevant specifications).

### 4.3. Management Implications

The IUCN extinction risk status is considered a credible index and source of information for conservation initiatives. However, field-based IUCN threat risk studies on selected plant taxa have been, and will probably continue to be, an ad hoc process depending upon current knowledge on specific, already assessed taxa, individual initiative, enthusiasm and subject to funding availability [111]. This evidently biases conservation efforts and national strategies against a holistic approach for plant diversity protection, since it lacks data on a larger scale in terms of the number of the assessed taxa, their spatial distribution and overlaps among them. Here we attempted to overcome these limitations and reduce bias, by providing a comprehensive catalogue for the IUCN extinction risk status of the entire Greek endemic flora which serves as a fundamental tool to inform conservation policy and decision makers for future strategies and management implementations in Greece. The drafting of this catalogue is considered a decision-making prerequisite and a cornerstone for environmental management, since plant diversity’s continuing decline will have a greater impact on human well-being than any other type of biodiversity loss [111], setting relevant conservation actions at the top of the policy agenda. For instance, this assessment could complement national-scale efforts on protecting valuable ecosystem services related to threatened taxa with ecological, commercial, and cultural importance, (e.g., *Lamiaceae* endemics [112]). By this, our results can support decision makers to adjust conservation actions to both proactive and reactive terms and by this optimally and efficiently allocate efforts and resources on biodiversity investment decisions. Apart from the need of a nationwide partial redesign of the Natura 2000 PA network [51,78] by e.g., establishing Key Biodiversity Areas [113,114,115] that might complement the existing PA network, further steps regarding conservation actions in Greece should include: (a) surveys on threatened taxa with respect to climate change, food security, genetic resources loss, ecosystem services loss, (b) active involvement of the National herbaria, creating the necessary bridge between taxonomic collections and conservation [111], and (c) dissemination of the scientific knowledge via the communication of the extinction risk impact on human prosperity and well-being, in order to reach the top of the local, regional and national policy agenda. Moreover, the information presented here can be incorporated in various prioritization schemes at local, national and global level (see also [116]). The supplementary provision of the spatial distribution data for taxa included in the catalogue, can provide direct input to the zonation methodology for the Natura 2000 PA network for the relevant area prioritization process, which is ongoing in Greece and deals with crucial trade-offs among land uses and human-nature interaction. Finally, by disseminating the extinction risk status of every Greek endemic taxon, conservation funds in Greece may be more appropriately allotted and not spent entirely in information-gathering as is the case for most recovery plans [117], but in conservation and protection actions or in certain cases, to serve as a baseline for more in-depth conservation assessments in the future.

## 5. Conclusions

Threatened species lists, as the one provided here, fulfil important political, social and scientific needs in biodiversity conservation. Nevertheless, it is naïve and counterproductive to use them in isolation when allocating resources for conservation [116,118]. The most frequent case is that decision making on conservation management deals with a variety of trade-offs including socio-economic, socio-cultural, and socio-ecological factors and thus all conservation goals can be rarely achieved simultaneously [119]. In the Anthropocene era, where climate-change and land-use change are projected to significantly alter the biodiversity patterns and may force many taxa to extinction, our assessment provides the baseline and acts as a guide for future conservation research and sustainable management. It might thus prove crucial for the timely, systematic, and effective aversion of plant extinctions in Greece and ignite the drafting of a new, comprehensive Red Data Book for the Greek flora.

## Data Availability

Not applicable.

## Supplementary Materials

The following are available online at https://www.mdpi.com/2079-7737/10/3/195/s1, Figure S1: Red coloring indicates grid cells with L1 (top 1%) values for the Greek endemic taxa. Dashed lines denote the protected areas present in Greece, Figure S2: Red coloring indicates grid cells with L1 (top 1%) values for the Critically Endangered Greek endemic taxa. Dashed lines denote the protected areas present in Greece, Table S1: The Greek endemic plant taxa, along with information on each taxon’s extinction risk status for every both IUCN Criteria A and B. EDGE: Evolutionary Distinct and Globally Endangered index. ERA: Extinction risk based on the IUCN Criterion A. ERB: Extinction risk based on the IUCN Criterion B. ERAB: Extinction risk based on both the IUCN Criteria A and B, Table S2: Families with 10 or more Greek endemic plant taxa characterized as Critically Endangered (CR) and the highest percentage of Critically Endangered taxa. ERAB: Extinction risk based on both the IUCN Criteria A and B. n: number of taxa. Percentages are shown in descending order, Table S3: Genera with 10 or more Greek endemic plant taxa characterized as Critically Endangered (CR) and the highest percentage of Critically Endangered taxa. ERAB: Extinction risk based on both the IUCN Criteria A and B. n: number of taxa. Percentages are shown in descending order, Table S4: The Greek endemic plant taxa characterized as Critically Endangered based on the IUCN Criterion A or B. ERA: Extinction risk based on the IUCN Criterion A. ERB: Extinction risk based on the IUCN Criterion B. AOO: Area of Occupancy. EOO: Extent of Occurrence. AOO_full: Area of Occupancy estimated from all occurrences. AOO_red: Area of Occupancy estimated only from occurrences situated outside human affected areas. AOO_decline: Estimated population reduction percentage for each taxon inferred by a potential decrease in the Area of Occupancy ([AOO_full—AOO_red]/AOO_full) × 100). Locations: Number of locations for every taxon based on occurrence records. All AOO and EOO metrics are given in square kilometres, Table S5: Confusion matrix results comparing the IUCN extinction risk status of 238 Greek endemic taxa based on occurrence data [i.e., the ConR and Stevart’s et al. (2019) approach we followed] against control threat categories [data derived from Phitos et al. (1995, 2009)] given as percentages. Regarding the accuracy metric, the confidence intervals are given in parentheses, Table S6: The fourteen Greek endemic plant taxa that should be prioritized in terms of conservation effort based on the EDGE index. EDGE: Evolutionary Distinct and Globally Endangered. ERAB: Extinction risk based on both the IUCN Criteria A and B. CR: Critically Endangered. EN: Endangered.

## References

[1] Lughadha E.N., Govaerts R., Belyaeva I., Black N., Lindon H., Allkin R., Magill R.E., Nicolson N. (2016). Counting counts: Revised estimates of numbers of accepted species of flowering plants, seed plants, vascular plants and land plants with a review of other recent estimates. Phytotaxa.

[2] Christenhusz M.J.M., Byng J.W. (2016). The number of known plants species in the world and its annual increase. Phytotaxa.

[3] Kier G., Kreft H., Lee T.M., Jetz W., Ibisch P.L., Nowicki C., Mutke J., Barthlott W. (2009). A global assessment of endemism and species richness across island and mainland regions. Proc. Natl. Acad. Sci. USA.

[4] Gallagher R.V., Allen S., Rivers M.C., Allen A.P., Butt N., Keith D., Auld T.D., Enquist B.J., Wright I.J., Possingham H.P. (2020). Global shortfalls in extinction risk assessments for endemic flora. bioRxiv.

[5] Barthlott W., Mutke J., Rafiqpoor D., Kier G., Kreft H. (2005). Global Centers of Vascular Plant Diversity. Nov. Acta Leopoldina.

[6] Myers N., Mittermeier R.A., Mittermeier C.G., Da Fonseca G.A.B., Kent J. (2000). Biodiversity hotspots for conservation priorities. Nature.

[7] Le Roux J.J., Hui C., Castillo M.L., Iriondo J.M., Keet J.H., Khapugin A.A., Médail F., Rejmánek M., Theron G., Yannelli F.A. (2019). Recent Anthropogenic Plant Extinctions Differ in Biodiversity Hotspots and Coldspots. Curr. Biol..

[8] Vellend M., Baeten L., Becker-Scarpitta A., Boucher-Lalonde V., McCune J.L., Messier J., Myers-Smith I.H., Sax D.F. (2017). Plant Biodiversity Change Across Scales During the Anthropocene. Annu. Rev. Plant Biol..

[9] Gray A. (2019). The ecology of plant extinction: Rates, traits and island comparisons. Oryx.

[10] Pimm S.L., Jenkins C.N., Abell R., Brooks T.M., Gittleman J.L., Joppa L.N., Raven P.H., Roberts C.M., Sexton J.O. (2014). The biodiversity of species and their rates of extinction, distribution, and protection. Science.

[11] Newbold T. (2018). Future effects of climate and land-use change on terrestrial vertebrate community diversity under different scenarios. Proc. R. Soc. B Biol. Sci..

[12] Newbold T., Hudson L.N., Contu S., Hill S.L.L., Beck J., Liu Y., Meyer C., Phillips H.R.P., Scharlemann J.P.W., Purvis A. (2018). Widespread winners and narrow-ranged losers: Land use homogenizes biodiversity in local assemblages worldwide. PLoS Biol..

[13] Powers R.P., Jetz W. (2019). Global habitat loss and extinction risk of terrestrial vertebrates under future land-use-change scenarios. Nat. Clim. Chang..

[14] Tsianou M.A., Touloumis K., Kallimanis A.S. (2021). Low spatial congruence between temporal functional β-diversity and temporal taxonomic and phylogenetic β-diversity in British avifauna. Ecol. Res..

[15] Cronk Q. (2016). Plant extinctions take time. Science.

[16] Rounsevell M.D.A., Harfoot M., Harrison P.A., Newbold T., Gregory R.D., Mace G.M. (2020). A biodiversity target based on species extinctions. Science.

[17] CBD (Convention on Biological Diversity) X/17. Consolidated update of the Global Strategy for Plant Conservation 2011–2020. https://www.cbd.int/kb/record/decision/12283?RecordType=decision.

[18] Corlett R.T. (2020). Safeguarding our future by protecting biodiversity. Plant Divers..

[19] Heywood V.H. (2019). Conserving plants within and beyond protected areas—Still problematic and future uncertain. Plant Divers..

[20] Bachman S.P., Field R., Reader T., Raimondo D., Donaldson J., Schatz G.E., Lughadha E.N. (2019). Progress, challenges and opportunities for Red Listing. Biol. Conserv..

[21] Heywood V.H. (2017). Plant conservation in the Anthropocene—Challenges and future prospects. Plant Divers..

[22] Nic Lughadha E., Bachman S.P., Leão T.C.C., Forest F., Halley J.M., Moat J., Acedo C., Bacon K.L., Brewer R.F.A., Gâteblé G. (2020). Extinction risk and threats to plants and fungi. Plants People Planet.

[23] Beck J., Ballesteros-Mejia L., Nagel P., Kitching I.J. (2013). Online solutions and the ‘Wallacean shortfall’: What does GBIF contribute to our knowledge of species’ ranges?. Divers. Distrib..

[24] Wilson E.O. (2017). Biodiversity research requires more boots on the ground: Comment. Nat. Ecol. Evol..

[25] Pressey R.L., Mills M., Weeks R., Day J.C. (2013). The plan of the day: Managing the dynamic transition from regional conservation designs to local conservation actions. Biol. Conserv..

[26] Pfab M.F., Victor J.E., Armstrong A.J. (2011). Application of the IUCN Red Listing system to setting species targets for conservation planning purposes. Biodivers. Conserv..

[27] Le Breton T.D., Zimmer H.C., Gallagher R.V., Cox M., Allen S., Auld T.D. (2019). Using IUCN criteria to perform rapid assessments of at-risk taxa. Biodivers. Conserv..

[28] Stévart T., Dauby G., Lowry P.P., Blach-Overgaard A., Droissart V., Harris D.J., Mackinder B.A., Schatz G.E., Sonké B., Sosef M.S.M. (2019). A third of the tropical African flora is potentially threatened with extinction. Sci. Adv..

[29] Miller J.S., Porter-Morgan H.A., Stevens H., Boom B., Krupnick G.A., Acevedo-Rodríguez P., Fleming J., Gensler M. (2012). Addressing target two of the Global Strategy for Plant Conservation by rapidly identifying plants at risk. Biodivers. Conserv..

[30] Bachman S.P., Nic Lughadha E.M., Rivers M.C. (2018). Quantifying progress toward a conservation assessment for all plants. Conserv. Biol..

[31] Walls S.C. (2018). Coping with constraints: Achieving effective conservation with limited resources. Front. Ecol. Evol..

[32] Drechsler M., Eppink F.V., Wätzold F. (2011). Does proactive biodiversity conservation save costs?. Biodivers. Conserv..

[33] Arponen A. (2012). Prioritizing species for conservation planning. Biodivers. Conserv..

[34] Daru B.H., le Roux P.C., Gopalraj J., Park D.S., Holt B.G., Greve M. (2019). Spatial overlaps between the global protected areas network and terrestrial hotspots of evolutionary diversity. Glob. Ecol. Biogeogr..

[35] Isaac N.J.B., Turvey S.T., Collen B., Waterman C., Baillie J.E.M. (2007). Mammals on the EDGE: Conservation Priorities Based on Threat and Phylogeny. PLoS ONE.

[36] Dauby G., Stévart T., Droissart V., Cosiaux A., Deblauwe V., Simo-Droissart M., Sosef M.S.M., Lowry P.P., Schatz G.E., Gereau R.E. (2017). ConR: An R package to assist large-scale multispecies preliminary conservation assessments using distribution data. Ecol. Evol..

[37] Lughadha E.N., Walker B.E., Canteiro C., Chadburn H., Davis A.P., Hargreaves S., Lucas E.J., Schuiteman A., Williams E., Bachman S.P. (2019). The use and misuse of herbarium specimens in evaluating plant extinction risks. Philos. Trans. R. Soc. B Biol. Sci..

[38] Dimopoulos P., Raus T., Bergmeier E., Constantinidis T., Iatrou G., Kokkini S., Strid A., Tzanoudakis D. (2013). Vascular plants of Greece: An annotated checklist. Englera.

[39] Phitos D., Constantinidis T.H., Kamari G. (2009). The Red Data Book of Rare and Threatened Plants of Greece.

[40] Phitos D., Strid A., Snogerup S., Greuter W. (1995). The Red Data Book of Rare and Threatened Plants of Greece.

[41] Kokkoris I.P., Mallinis G., Bekri E.S., Vlami V., Zogaris S., Chrysafis I., Mitsopoulos I., Dimopoulos P. (2020). National Set of MAES Indicators in Greece: Ecosystem Services and Management Implications. Forests.

[42] Hortal J., de Bello F., Diniz-Filho J.A.F., Lewinsohn T.M., Lobo J.M., Ladle R.J. (2015). Seven Shortfalls that Beset Large-Scale Knowledge of Biodiversity. Annu. Rev. Ecol. Evol. Syst..

[43] Strid A. (2016). Atlas of the Aegean Flora.

[44] Strid A. (1996). Phytogeographia Aegaea and the flora Hellenica database. Ann. Naturhistorischen Museums Wien. Ser. B für Bot. Zool..

[45] Kougioumoutzis K., Valli A.T., Georgopoulou E., Simaiakis S.M., Triantis K.A., Trigas P. (2017). Network biogeography of a complex island system: The Aegean Archipelago revisited. J. Biogeogr..

[46] Kougioumoutzis K., Simaiakis S.M., Tiniakou A. (2014). Network biogeographical analysis of the central Aegean archipelago. J. Biogeogr..

[47] Jin Y., Qian H.V. (2019). PhyloMaker: An R package that can generate very large phylogenies for vascular plants. Ecography.

[48] Smith S.A., Brown J.W. (2018). Constructing a broadly inclusive seed plant phylogeny. Am. J. Bot..

[49] Dimopoulos P., Raus T., Bergmeier E., Constantinidis T., Iatrou G., Kokkini S., Strid A., Tzanoudakis D. (2016). Vascular plants of Greece: An annotated checklist. Supplement. Willdenowia.

[50] QGIS Development Team (2020). QGIS Geographic Information System.

[51] Kougioumoutzis K., Kokkoris I.P., Panitsa M., Kallimanis A., Strid A., Dimopoulos P. (2021). Plant Endemism Centres and Biodiversity Hotspots in Greece. Biology.

[52] Dauby G. ConR: Computation of Parameters Used in Preliminary Assessment of Conservation Status 2017. https://www.researchgate.net/project/RAINBIO.

[53] Zizka A., Azevedo J., Leme E., Neves B., da Costa A.F., Caceres D., Zizka G. (2020). Biogeography and conservation status of the pineapple family (Bromeliaceae). Divers. Distrib..

[54] Zizka A., Silvestro D., Vitt P., Knight T.M. (2020). Automated conservation assessment of the orchid family with deep learning. Conserv. Biol..

[55] IUCN (2019). IUCN Standards and Petitions Committee Guidelines for Using the IUCN Red List Categories and Criteria.

[56] Collen B., Dulvy N.K., Gaston K.J., Gärdenfors U., Keith D.A., Punt A.E., Regan H.M., Böhm M., Hedges S., Seddon M. (2016). Clarifying misconceptions of extinction risk assessment with the IUCN Red List. Biol. Lett..

[57] Brummitt N., Bachman S.P., Aletrari E., Chadburn H., Griffiths-Lee J., Lutz M., Moat J., Rivers M.C., Syfert M.M., Nic Lughadha E.M. (2015). The Sampled Red List Index for Plants, phase II: Ground-truthing specimen-based conservation assessments. Philos. Trans. R. Soc. B Biol. Sci..

[58] Ballantyne M., Pickering C.M. (2013). Tourism and recreation: A common threat to IUCN red-listed vascular plants in Europe. Biodivers. Conserv..

[59] Bergmeier E., Strid A. (2014). Regional diversity, population trends and threat assessment of the weeds of traditional agriculture in Greece. Bot. J. Linn. Soc..

[60] García-Vega D., Newbold T. (2020). Assessing the effects of land use on biodiversity in the world’s drylands and Mediterranean environments. Biodivers. Conserv..

[61] Kougioumoutzis K., Tiniakou A. (2014). Ecological factors driving plant species diversity in the South Aegean Volcanic Arc and other central Aegean islands. Plant Ecol. Divers..

[62] Panitsa M., Kagiampaki A., Kougioumoutzis K., Sfenthourakis S., Pafilis P., Parmakelis A., Poulakakis N., Triantis K. (2018). Plant diversity and biogeography of the Aegean Archipelago: A New Synthesis. Biogeography and Biodiversity of the Aegean. In honour of Prof. Moysis Mylonas.

[63] Panitsa M., Iliadou E., Kokkoris I., Kallimanis A., Patelodimou C., Strid A., Raus T., Bergmeier E., Dimopoulos P. (2020). Distribution patterns of ruderal plant diversity in Greece. Biodivers. Conserv..

[64] Rivers M.C., Brummitt N.A., Nic Lughadha E., Meagher T.R. (2014). Do species conservation assessments capture genetic diversity?. Glob. Ecol. Conserv..

[65] Panter C.T., Clegg R.L., Moat J., Bachman S.P., Klitgård B.B., White R.L. (2020). To clean or not to clean: Cleaning open-source data improves extinction risk assessments for threatened plant species. Conserv. Sci. Pract..

[66] Kuhn M. (2008). Building predictive models in R using the caret package. J. Stat. Softw..

[67] Altman D.G., Bland J.M. (1994). Statistics Notes: Diagnostic tests 1: Sensitivity and specificity. BMJ.

[68] Altman D.G., Bland J.M. (1994). Statistics Notes: Diagnostic tests 2: Predictive values. BMJ.

[69] Velez D.R., White B.C., Motsinger A.A., Bush W.S., Ritchie M.D., Williams S.M., Moore J.H. (2007). A balanced accuracy function for epistasis modeling in imbalanced datasets using multifactor dimensionality reduction. Genet. Epidemiol..

[70] Hastie T., Tibshirani R., Friedman J. (2009). The Elements of Statistical Learning: Data Mining, Inference, and Prediction.

[71] Kuhn M. (2020). Caret: Classification and Regression Training. R Package Version 6.0-86. https://cran.r-project.org/web/packages/crtests/vignettes/overview.html.

[72] González-Orozco C.E., Laffan S.W., Miller J.T. (2011). Spatial distribution of species richness and endemism of the genus Acacia in Australia. Aust. J. Bot..

[73] Cañadas E.M., Fenu G., Peñas J., Lorite J., Mattana E., Bacchetta G. (2014). Hotspots within hotspots: Endemic plant richness, environmental drivers, and implications for conservation. Biol. Conserv..

[74] IUCN (2010). Guidelines for Application of Iucn Red List Criteria At Guidelines for Application of Iucn Red List Criteria At Regional and National Levels.

[75] Kembel S.W., Cowan P.D., Helmus M.R., Cornwell W.K., Morlon H., Ackerly D.D., Blomberg S.P., Webb C.O. (2010). Picante: R tools for integrating phylogenies and ecology. Bioinformatics.

[76] Hanson J. wdpar: Interface to the World Database on Protected Areas. R Package Version 1.0.5 2020. https://www.iucn.org/theme/protected-areas/our-work/quality-and-effectiveness/world-database-protected-areas-wdpa#:~:text=The%20World%20Database%20on%20Protected,in%20conserving%20species%20and%20ecosystems.

[77] Pebesma E. (2018). Simple features for R: Standardized support for spatial vector data. R J..

[78] Spiliopoulou K., Dimitrakopoulos P.G., Brooks T.M., Kelaidi G., Paragamian K., Kati V., Oikonomou A., Vavylis D., Trigas P., Lymberakis P. (2021). The Natura 2000 network and the ranges of threatened species in Greece. Biodivers. Conserv..

[79] Orsenigo S., Montagnani C., Fenu G., Gargano D., Peruzzi L., Abeli T., Alessandrini A., Bacchetta G., Bartolucci F., Bovio M. (2018). Red Listing plants under full national responsibility: Extinction risk and threats in the vascular flora endemic to Italy. Biol. Conserv..

[80] Muñoz-Rodríguez P., Draper Munt D., Moreno Saiz J.C. (2016). Global strategy for plant conservation: Inadequate in situ conservation of threatened flora in Spain. Isr. J. Plant Sci..

[81] Brundu G., Peruzzi L., Domina G., Bartolucci F., Galasso G., Peccenini S., Raimondo F.M., Albano A., Alessandrini A., Banfi E. (2017). At the intersection of cultural and natural heritage: Distribution and conservation of the type localities of Italian endemic vascular plants. Biol. Conserv..

[82] Huang J., Huang J., Liu C., Zhang J., Lu X., Ma K. (2016). Diversity hotspots and conservation gaps for the Chinese endemic seed flora. Biol. Conserv..

[83] Koutroumpa K., Theodoridis S., Warren B.H., Jiménez A., Celep F., Doğan M., Romeiras M.M., Santos-Guerra A., Fernández-Palacios J.M., Caujapé-Castells J. (2018). An expanded molecular phylogeny of Plumbaginaceae, with emphasis on Limonium (sea lavenders): Taxonomic implications and biogeographic considerations. Ecol. Evol..

[84] Koutroumpa K., Warren B.H., Theodoridis S., Coiro M., Romeiras M.M., Jiménez A., Conti E. (2020). Geo-climatic changes and apomixis as major drivers of diversification in the Mediterranean sea lavenders (Limonium Mill.). Front. Plant Sci..

[85] Newbold T., Oppenheimer P., Etard A., Williams J.J. (2020). Tropical and Mediterranean biodiversity is disproportionately sensitive to land-use and climate change. Nat. Ecol. Evol..

[86] Doxa A., Albert C.H., Leriche A., Saatkamp A. (2017). Prioritizing conservation areas for coastal plant diversity under increasing urbanization. J. Environ. Manag..

[87] Troia A., Greuter W. (2015). A conspectus of and key to Greek Isoetes (Isoetaceae), based on a reassessment of Haussknecht’s gatherings of 1885. Willdenowia.

[88] Constantinidis T., Kalpoutzakis E., Kougioumoutzis K. (2015). The rediscovery of Stachys virgata (Lamiaceae), a rare endemic of Peloponnisos, Greece: Taxonomy, distribution, karyology and conservation. Phytotaxa.

[89] Yannitsaros A.G., Constantinidis T.A., Vassiliades D.D. (1996). The rediscovery of Biebersteinia orphanidis Boiss. (Geraniaceae) in Greece. Bot. J. Linn. Soc..

[90] Aedo C., Medina L., Barberá P., Fernández-Albert M. (2015). Extinctions of vascular plants in Spain. Nord. J. Bot..

[91] Rejmánek M. (2018). Vascular plant extinctions in California: A critical assessment. Divers. Distrib..

[92] Strid A. (2020). The botanical exploration of Greece. Plant Syst. Evol..

[93] Halley J.M., Monokrousos N., Mazaris A.D., Vokou D. (2017). Extinction debt in plant communities: Where are we now?. J. Veg. Sci..

[94] Kopsidis M., Ivanov M. (2017). Industrialization and De-industrialization in Southeast Europe, 1870–2010. Spread Mod. Ind. Peripher. Since 1871.

[95] Gomellini M., Toniolo G. (2017). The Industrialization of Italy, 1861–1971. Spread Mod. Ind. Peripher. Since 1871.

[96] Tsani S.Z. (2010). Energy consumption and economic growth: A causality analysis for Greece. Energy Econ..

[97] Kougioumoutzis K., Kokkoris I.P., Panitsa M., Trigas P., Strid A., Dimopoulos P. (2020). Plant Diversity Patterns and Conservation Implications under Climate-Change Scenarios in the Mediterranean: The Case of Crete (Aegean, Greece). Diversity.

[98] Fassou G., Kougioumoutzis K., Iatrou G., Trigas P., Papasotiropoulos V. (2020). Genetic Diversity and Range Dynamics of Helleborus odorus subsp. cyclophyllus under Different Climate Change Scenarios. Forests.

[99] Stathi E., Kougioumoutzis K., Abraham E.M., Trigas P., Ganopoulos I., Avramidou E.V., Tani E. (2020). Population genetic variability and distribution of the endangered Greek endemic Cicer graecum under climate change scenarios. AoB Plants.

[100] Tsiftsis S., Djordjević V. (2020). Modelling sexually deceptive orchid species distributions under future climates: The importance of plant–pollinator interactions. Sci. Rep..

[101] Kougioumoutzis K., Kokkoris I.P., Panitsa M., Trigas P., Strid A., Dimopoulos P. (2020). Spatial Phylogenetics, Biogeographical Patterns and Conservation Implications of the Endemic Flora of Crete (Aegean, Greece) under Climate Change Scenarios. Biology.

[102] Tucker C.M., Cadotte M.W. (2013). Unifying measures of biodiversity: Understanding when richness and phylogenetic diversity should be congruent. Divers. Distrib..

[103] Humphreys A.M., Govaerts R., Ficinski S.Z., Nic Lughadha E., Vorontsova M.S. (2019). Global dataset shows geography and life form predict modern plant extinction and rediscovery. Nat. Ecol. Evol..

[104] Paliogiannis C., Koedam N., Cliquet A. (2019). The impact of the economic crisis on the implementation of the EU Nature Directives in Greece: An expert-based view. J. Nat. Conserv..

[105] Gittenberger E. (1991). What about non-adaptive radiation?. Biol. J. Linn. Soc..

[106] Maes J., Teller A., Erhard M., Grizzetti B., Barredo J.I., Paracchini M.-L., Condé S., Somma F., Orgiazzi A., Jones A. (2018). Mapping and Assessment of Ecosystems and their Services: An Analytical Framework for Ecosystem Condition.

[107] Müller A., Schneider U.A., Jantke K. (2020). Evaluating and expanding the European Union’s protected-area network toward potential post-2020 coverage targets. Conserv. Biol..

[108] Allan J.R., Possingham H.P., Atkinson S.C., Waldron A., Di Marco M., Adams V.M., Butchart S.H.M., Venter O., Maron M., Williams B.A. (2019). Conservation attention necessary across at least 44% of Earth’s terrestrial area to safeguard biodiversity. bioRxiv.

[109] Dudley N. (2008). Guidelines for Applying Protected Area Management Categories.

[110] International Union for Conservation of Nature (IUCN) (2019). Recognising and Reporting Other Effective Area-Based Conservation Measures.

[111] Schatz G.E. (2009). Plants on the IUCN Red List: Setting priorities to inform conservation. Trends Plant Sci..

[112] Cheminal A., Kokkoris I.P., Strid A., Dimopoulos P. (2020). Medicinal and Aromatic Lamiaceae Plants in Greece: Linking Diversity and Distribution Patterns with Ecosystem Services. Forests.

[113] IUCN (2016). A Global Standard for the Identification of Key Biodiversity Areas, Version 1.0.

[114] Eken G., Bennun L., Brooks T.M., Darwall W., Fishpool L.D.C., Foster M., Knox D., Langhammer P., Matiku P., Radford E. (2004). Key biodiversity areas as site conservation targets. Bioscience.

[115] Brooks T.M., Cuttelod A., Faith D.P., Garcia-Moreno J., Langhammer P., Pérez-Espona S. (2015). Why and how might genetic and phylogenetic diversity be reflected in the identification of key biodiversity areas?. Philos. Trans. R. Soc. B Biol. Sci..

[116] Liu U., Kenney S., Breman E., Cossu T.A. (2019). A multicriteria decision making approach to prioritise vascular plants for species-based conservation. Biol. Conserv..

[117] Buxton R.T., Avery-Gomm S., Lin H.Y., Smith P.A., Cooke S.J., Bennett J.R. (2020). Half of resources in threatened species conservation plans are allocated to research and monitoring. Nat. Commun..

[118] Possingham H.P., Andelman S.J., Burgman M.A., Medellĺn R.A., Master L.L., Keith D.A. (2002). Limits to the use of threatened species lists. Trends Ecol. Evol..

[119] Pearse W.D., Chase M.W., Crawley M.J., Dolphin K., Fay M.F., Joseph J.A., Powney G., Preston C.D., Rapacciuolo G., Roy D.B. (2015). Beyond the EDGE with EDAM: Prioritising British Plant Species According to Evolutionary Distinctiveness, and Accuracy and Magnitude of Decline. PLoS ONE.

