# Supplementary material for: Extinction Risk Assessment of the Greek Endemic Flora
Source: Biology (Basel). 2021 Mar 4;10(3):195. doi: 10.3390/biology10030195 (PMC7999807; doi:10.3390/biology10030195)
Supplement: Supplementary file 1 [file biology-10-00195-s001.zip › Supplementary Tables & Figures.docx]

**Extinction risk assessment of the Greek endemic flora**

Biology

Supplementary tables and figures

**Supplementary tables**

**Table S2.** Families with 10 or more Greek endemic plant taxa characterised as Critically Endangered (CR) and the highest percentage of Critically Endangered taxa. ERAB: Extinction risk based on both the IUCN Criteria A and B. n: number of taxa. Percentages are shown in descending order.

| **Family** | **ERAB** | **n** | **%** |
| --- | --- | --- | --- |
| Violaceae | CR | 18 | 78.3 |
| Apiaceae | CR | 30 | 65.2 |
| Asteraceae | CR | 164 | 58.0 |
| Scrophulariaceae | CR | 15 | 51.7 |
| Alliaceae | CR | 24 | 50.0 |
| Brassicaceae | CR | 43 | 48.9 |
| Caryophyllaceae | CR | 73 | 47.1 |
| Poaceae | CR | 12 | 46.2 |
| Lamiaceae | CR | 39 | 37.9 |
| Rubiaceae | CR | 23 | 37.1 |
| Plumbaginaceae | CR | 30 | 35.7 |
| Boraginaceae | CR | 14 | 34.1 |
| Fabaceae | CR | 15 | 33.3 |
| Campanulaceae | CR | 17 | 26.2 |

**Table S3.** Genera with 10 or more Greek endemic plant taxa characterised as Critically Endangered (CR) and the highest percentage of Critically Endangered taxa. ERAB: Extinction risk based on both the IUCN Criteria A and B. n: number of taxa. Percentages are shown in descending order.

| **Family** | **Genus** | **ERAB** | **n** | **%** |
| --- | --- | --- | --- | --- |
| Asteraceae | *Hieracium* | CR | 51 | 85.0 |
| Caryophyllaceae | *Minuartia* | CR | 10 | 83.3 |
| Violaceae | *Viola* | CR | 18 | 78.3 |
| Asteraceae | *Crepis* | CR | 10 | 76.9 |
| Asteraceae | *Taraxacum* | CR | 24 | 64.9 |
| Brassicaceae | *Alyssum* | CR | 10 | 58.8 |
| Alliaceae | *Allium* | CR | 24 | 50.0 |
| Asteraceae | *Centaurea* | CR | 39 | 50.0 |
| Scrophulariaceae | *Verbascum* | CR | 14 | 50.0 |
| Caryophyllaceae | *Silene* | CR | 24 | 44.4 |
| Rubiaceae | *Galium* | CR | 11 | 37.9 |
| Plumbaginaceae | *Limonium* | CR | 28 | 36.4 |
| Rubiaceae | *Asperula* | CR | 11 | 35.5 |
| Caryophyllaceae | *Dianthus* | CR | 11 | 31.4 |
| Campanulaceae | *Campanula* | CR | 17 | 28.3 |

**Table S5.** Confusion matrix results comparing the IUCN extinction risk status of 238 Greek endemic taxa based on occurrence data (i.e., the ConR and Stevart’s *et al*. [1] approach we followed) against control threat categories (data derived from Phitos *et al*. [2,3]) given as percentages. Regarding the accuracy metric, the confidence intervals are given in parentheses.

| **Criterion** | **Accuracy** | **Sensitivity** |
| --- | --- | --- |
| A | 65.1 (58.7 - 71.2) | 79.1 |
| B | 82.4 (76.9 - 87.0) | 92.4 |
| A & B | 80.7 (75.1 - 85.5) | 98 |

**Table S6.** The fourteen Greek endemic plant taxa that should be prioritized in terms of conservation effort based on the EDGE index. EDGE: Evolutionary Distinct and Globally Endangered. ERAB: Extinction risk based on both the IUCN Criteria A and B. CR: Critically Endangered. EN: Endangered.

| **Family** | **Genus** | **Taxon** | **EDGE** | **ERAB** |
| --- | --- | --- | --- | --- |
| Isoetaceae | *Isoetes* | *Isoetes heldreichii* | 8.77 | CR |
| Aspleniaceae | *Asplenium* | *Asplenium creticum* | 8.74 | CR |
| Pinaceae | *Abies* | *Abies cephalonica* | 7.87 | EN |
| Onagraceae | *Epilobium* | *Epilobium vernonicum* | 7.50 | CR |
| Saxifragaceae | *Saxifraga* | *Saxifraga sibthorpii* | 7.44 | CR |
| Tamaricaceae | *Tamarix* | *Tamarix minoa* | 7.32 | CR |
| Rhamnaceae | *Rhamnus* | *Rhamnus sibthorpiana* | 7.32 | CR |
| Ulmaceae | *Zelkova* | *Zelkova abelicea* | 7.32 | CR |
| Aceraceae | *Acer* | *Acer hyrcanum* subsp. *reginae-amaliae* | 7.31 | CR |
| Rosaceae | *Sanguisorba* | *Sanguisorba cretica* | 7.22 | CR |
| Santalaceae | *Thesium* | *Thesium vlachorum* | 7.16 | CR |
| Rosaceae | *Potentilla* | *Potentilla kionaea* | 7.12 | CR |
| Rosaceae | *Potentilla* | *Potentilla deorum* | 7.07 | CR |
| Rosaceae | *Alchemilla* | *Alchemilla aroanica* | 7.04 | CR |

**References**

1. Stévart, T.; Dauby, G.; Lowry, P.P.; Blach-Overgaard, A.; Droissart, V.; Harris, D.J.; Mackinder, B.A.; Schatz, G.E.; Sonké, B.; Sosef, M.S.M.; et al. A third of the tropical African flora is potentially threatened with extinction. *Sci. Adv.* **2019**, *5*, eaax9444, doi:10.1126/sciadv.aax9444.

2. Phitos, D.; Constantinidis, T.H.; Kamari, G. The red data book of rare and threatened plants of Greece. *Hell. Bot. Soc. Patras, Greece* **2009**.

3. Phitos, D.; Strid, A.; Snogerup, S.; Greuter, W. *The red data book of rare and threatened plants of Greece*; World Wide Fund for Nature, 1995; ISBN 9789607506047.

**Supplementary Figures**





**Figure S1.** Histogram of EDGE scores for the Greek endemic taxa, by threat category.


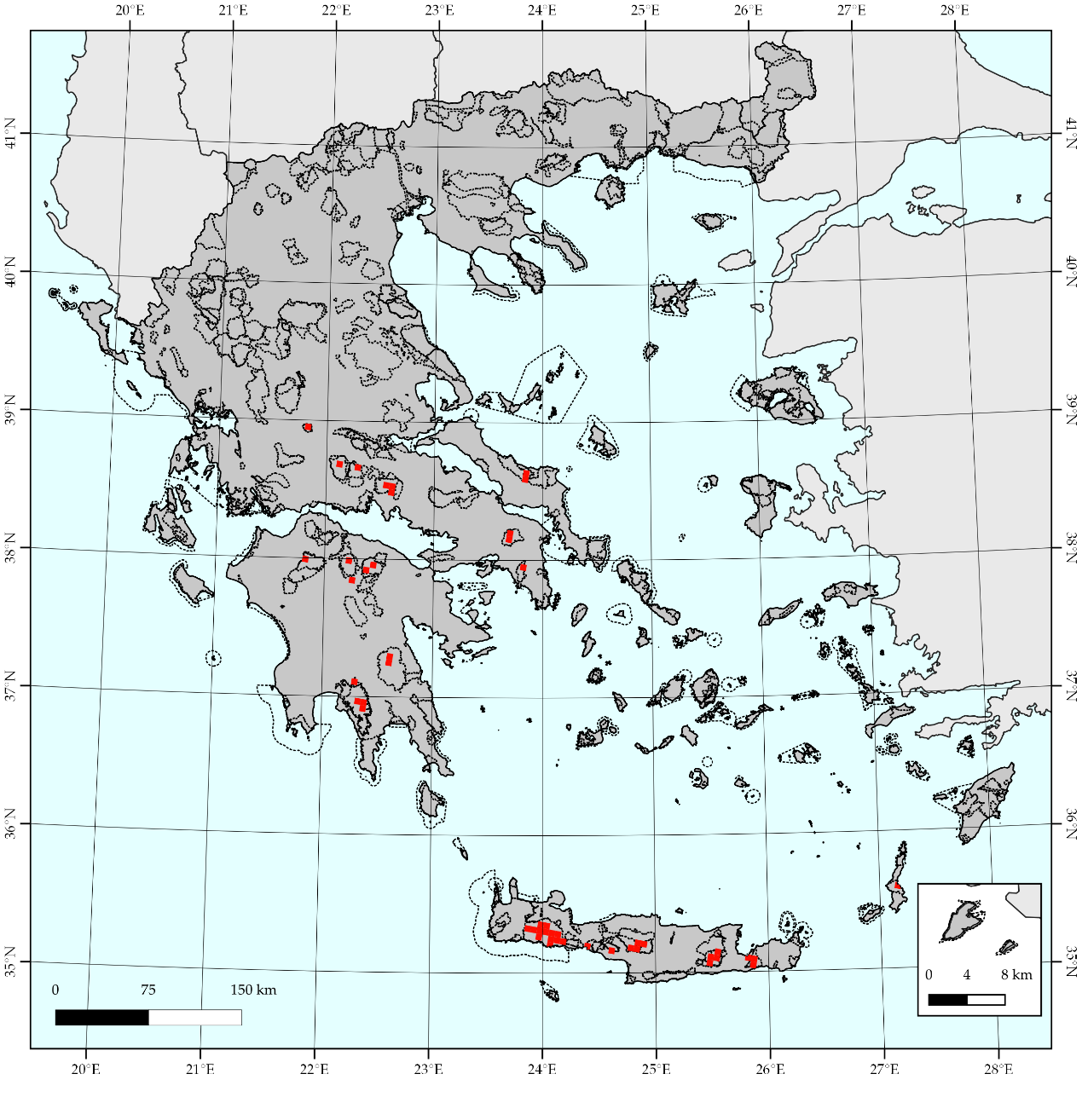


**Figure S2.** Red colouring indicates grid cells with L1 (top 1%) values for the Greek endemic taxa. Dashed lines denote the protected areas present in Greece. The inset map depicts the island of Megisti (Kastelorizo) and its nearby islets.

**
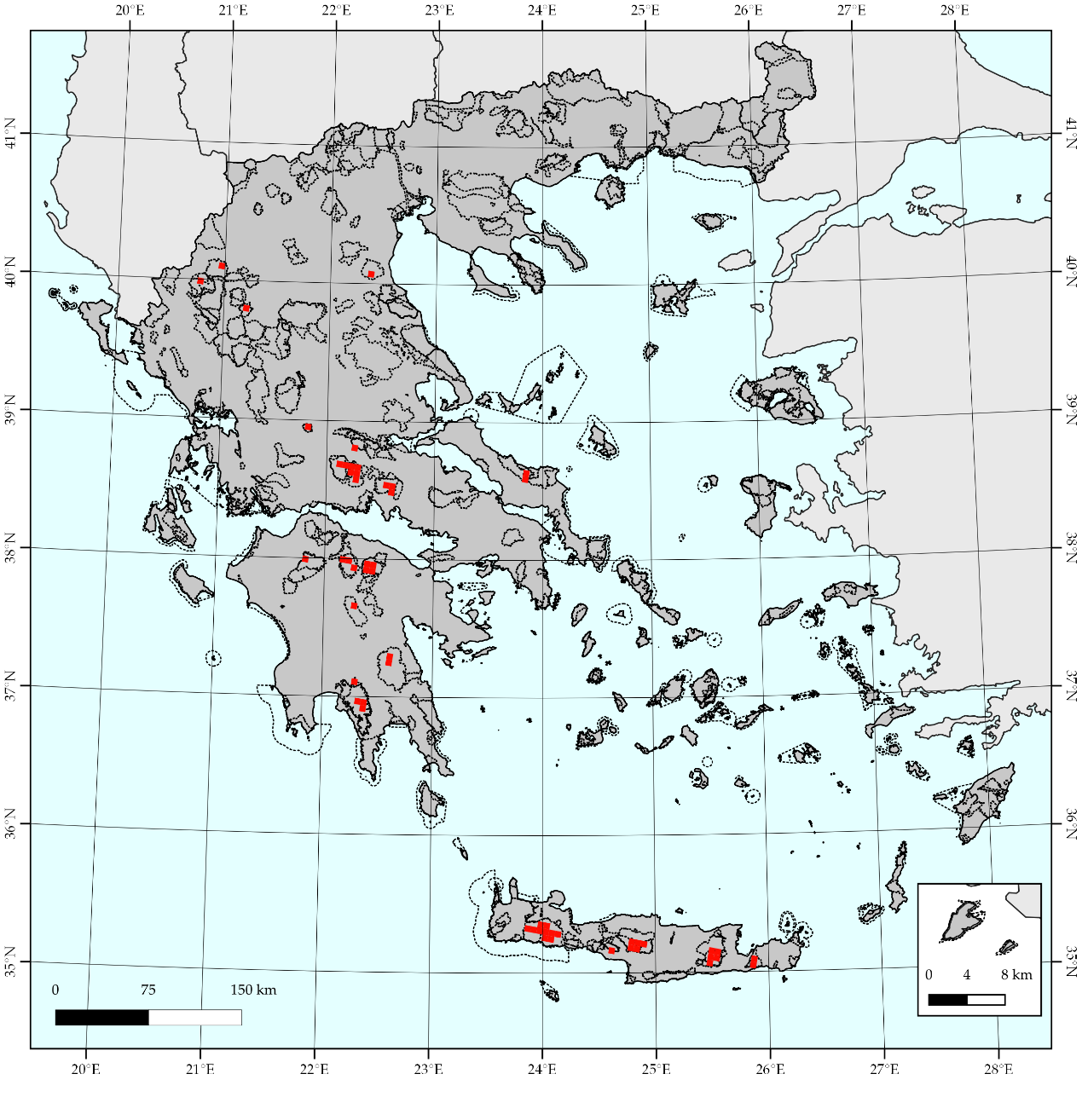
**

**Figure S3.** Red colouring indicates grid cells with L1 (top 1%) values for the Critically Endangered Greek endemic taxa. Dashed lines denote the protected areas present in Greece. The inset map depicts the island of Megisti (Kastelorizo) and its nearby islets.
